# Sighs Shape Respiratory Variability and Pupil Dynamics and Adapt to Sustained Attention Demands

**DOI:** 10.1111/psyp.70245

**Published:** 2026-01-23

**Authors:** Ralph W. G. Andrews, Michael C. Melnychuk, Paul M. Dockree

**Affiliations:** ^1^ Trinity Institute of Neuroscience (TCIN), Trinity College Dublin Dublin Ireland

## Abstract

Sighs are spontaneous deep breaths thought to play a homeostatic role in respiratory control. Their relationship to respiratory variability has been repeatedly demonstrated. How sighs are related to task engagement, performance, structure and arousal has remained unclear. Presently, we investigated sigh behavior across two sustained attention tasks using respiratory belt recordings. Participants completed either a Gradual Contrast Change Detection task (dataset Grad) or a Paced Auditory Cue Entrainment task (dataset PACE), with subgroups performing the latter under spontaneous (NIB) or slow‐paced (IB) breathing conditions. Sighs were identified as breaths at least twice the mean inspiratory volume (Vi). We analysed the total variability (coefficient of variation; CV) and structured variability (lag‐1 autocorrelation; AR) of respiratory rate (RR) and Vi, their changes over the task, and around sigh events. In spontaneous breathing groups (Grad, NIB), sigh frequency was positively related to CV in both RR and Vi suggesting a relationship to overall variability, and negatively correlated to RR‐AR, suggesting a relationship to the structure of the variability. Sigh frequency and CV increased over the task duration, while post‐sigh dynamics showed decreased CV and increased Vi‐AR, supporting sighs role in resetting the temporal structure. In IB group, sigh frequency was drastically reduced and no pre‐post sigh changes were observed. Sighs were also associated with changes in pupil diameter, implicating involvement of the noradrenaline‐mediated arousal system. Sighs were not related to any alterations in task performance or subjective engagement. Finally, stronger respiratory phase‐locking to task timing was associated with higher sigh frequency and increased respiratory variability, suggesting that sigh behavior could be influenced by task dynamics. The lack of task performance differences should be clarified using demanding tasks which could draw out variability. These findings support a role of sighs with respect to respiratory variability, phase‐locking behavior and pupil‐linked arousal during prolonged cognitive tasks.

## Abstract 2

This study strengthens existing evidence that sighs are closely linked to respiratory variability, extending prior work by showing that sighs reset both random and structured variability during sustained attention. We reveal a novel association between sigh frequency and respiratory phase‐locking to task dynamics, suggesting that sighs support adaptive regulation of cognitive–respiratory coupling. These findings offer insight into the mechanisms of breath‐based self‐regulation and have implications for therapeutic breathing practices targeting arousal and attention.

## 1 Introduction

The spontaneous respiratory rhythm is intermixed with breaths which are larger in volume than the average breath. These breaths are referred to in the literature as “sighs”, and are believed to have a fundamental role in maintaining healthy respiratory dynamics (Vlemincx, Van Diest, et al. 2010).

Spontaneous respiration is not constant in terms of rate and volume, but contains considerable variability which is thought to facilitate flexibility in the system, allowing it to adapt to changing states (Vlemincx, Van Diest, et al. 2010). For example, respiration adapts to different arousal (Balban et al. 2023; Shea 1996; Yackle et al. 2017) and emotional states (Boiten et al. 1994; Jerath and Beveridge 2020), and more recently, it has become increasingly recognized that respiration synchronizes its rhythm to sensory‐cognitive demands in a wide‐ranging manner (Grund et al. 2022; Huijbers et al. 2014; Johannknecht and Kayser 2022; Melnychuk et al. 2018; Perl et al. 2019).

Vlemincx and colleagues demonstrate that sighs can be viewed as a component within a dynamic respiratory system which acts as both a physiological (Severs et al. 2022) and psychological (Vlemincx et al. 2022) resetter. Physiologically, sighs reset variability in respiratory rate or volume; they counteract atelectasis (alveoli collapse) and are linked to arousal state transitions. Psychologically, sighs appear to occur during stressful scenarios and induce feelings of relief.

A large focus has been on the corrective action that sighs take to reset respiratory variability. Optimal respiratory variability is that which can carry out the fundamental gas exchange function but also maintain adaptability to changing states. For example, a “non‐stressful” sustained attention task was associated with participants having low respiratory variability and an increased sigh frequency in the subsequent recovery period. By contrast, when participants performed a “stressful” mental arithmetic task, their respiratory variability was high and sigh frequency also increased compared to rest (Vlemincx et al. 2011, 2012a). Both spontaneous and instructed sighs after these tasks had an effect of resetting the variability (Vlemincx et al. 2012a).

The authors distinguish between “structured variability” and “random variability”, with the former represented by the degree of autocorrelation in respiration at one‐breath lag and the latter inferred from the remaining unexplained portion of “total variability” (coefficient of variation). This distinction builds on foundational work by Daubenspeck & Bruce, and Tobin et al., who first proposed that breath‐to‐breath variability is not purely random but structured, reflecting central and peripheral feedback processes involved in respiratory control (Bruce 1996; Bruce and Daubenspeck 1995; Tobin et al. 1983a, 1983b). Vlemincx and colleagues (2010) found that sighs occur after a buildup of random respiratory variability and serve to restore structure to the variability, thereby rebalancing the system's variability profile and returning it to baseline.

As well as resetting respiratory variability, it is thought that sighs have a role in facilitating shifts between arousal states (Ramirez 2014; Severs et al. 2022). The evidence for this is largely through observing sighs around arousal behaviors during sleep (Perez‐Padilla et al. 1983; Ramirez et al. 2013), as well as possible linkages to the locus coeruleus noradrenaline system (LC‐NA) (Severs et al. 2022; Viemari et al. 2013). Akin to the resetting of variability, sighs may also reset arousal, as it has been seen that sighing increased in response to high‐arousal negatively valanced images (Vlemincx et al. 2015). Relatedly, the psychological counterpart to these physiological resets appears to be the experience of relief (Vlemincx et al. 2009, 2016; Vlemincx, Taelman, et al. 2010).

Research from our own group has investigated a relationship between respiration and attention from the perspective that they are coupled oscillators, interacting via the LC‐NA system (Andrews, Melnychuk, Moran, Walsh, et al. 2025; Andrews, Melnychuk, Moran, McGovern, et al. 2025; Melnychuk et al. 2018, 2021). This dynamical system model posits respiration, LC‐NA, and cortical attention networks as three sources of oscillations capable of influencing the relative stability in each other. This fluctuating synchronization and decoupling is proposed to contribute to producing emergent states of attention and the transitions between them. Within this model, deviations from a typical respiratory variability range would discourage inter‐oscillatory harmony between respiration and the LC‐NA and cortical attention systems, thus perhaps leading to suboptimal arousal and attentional states for the current context. A sigh here would not only act to reset respiratory variability, but would consequently facilitate synchronization with the arousal and attentional systems, perhaps leading to a more stable attentional state.

In the present paper, we sought to analyze sigh behavior in two previously collected datasets, pertaining to two different sustained attention tasks—the Gradual Contrast Change Detection—Experience Sampling task (dataset Grad) (Andrews, Melnychuk, Moran, Walsh, et al. 2025), and the Paced Auditory Cue Entrainment task (dataset PACE) (Andrews, Melnychuk, Moran, McGovern, et al. 2025). In light of the discussed prior work on sighs, showing that respiratory variability and sigh behavior are altered by attentional tasks (Vlemincx et al. 2011, 2012a), we saw opportunities to corroborate these previous findings and extend them from the perspective of a respiratory‐attentional coupling.

### 1.1 Dataset Grad

The GradCCD‐ES task requires participants to monitor a visual stimulus that intermittently decreases in contrast, and to report the detection of contrast changes with a button press. We found that participants exhibited respiratory phase‐locking to the events in the sustained attention task (Andrews, Melnychuk, Moran, Walsh, et al. 2025). Specifically, we found a majority group finding that participants synchronized their respiratory cycle with the occurrence of target presentations, such that there was a phase preference to experience them towards the latter portion of exhalation. That participants show an inhale/exhale preference for certain task events has been reported across a wide range of paradigms (Johannknecht and Kayser 2022), including mental imagery (Perl et al. 2019), tactile perception (Grund et al. 2022), and auditory oddball (Melnychuk et al. 2018) and visual lexical (Huijbers et al. 2014).

This task (GradCCD‐ES) was monotonous and of low demand and thus we could expect to see low respiratory variability concomitant with frequent sigh behavior, based on earlier findings by Vlemincx et al. (2011, 2012a). However, participants phase‐locked their respiratory rhythm to task events of variably changing inter‐presentation intervals (pseudo‐randomly 3, 5, or 7 s), and thus, this should have induced considerable random variability into their respiration. Sighs may therefore increase in frequency here, to reset variability, to maintain adaptability, and facilitate further phase‐locking. Either way, we should expect to see frequent sigh behavior and we intend to investigate here whether a sigh resets the respiratory variability, corroborating previous findings.

From the perspective of a respiratory‐attentional coupling, and an association between sighs, arousal, and relief, we further hypothesize that participants' attention to task could be “reset”. We intend to test this by comparing experience‐sampling thought probe responses (asking whether having task‐related or task‐unrelated thoughts) as well as reaction times either side of sighs for an indication of subjective and objective task focus, respectively. To our knowledge, no prior studies have tested for a pre‐ to post‐sigh difference in task performance or experiential focus.

### 1.2 Dataset PACE

**FIGURE 1:**
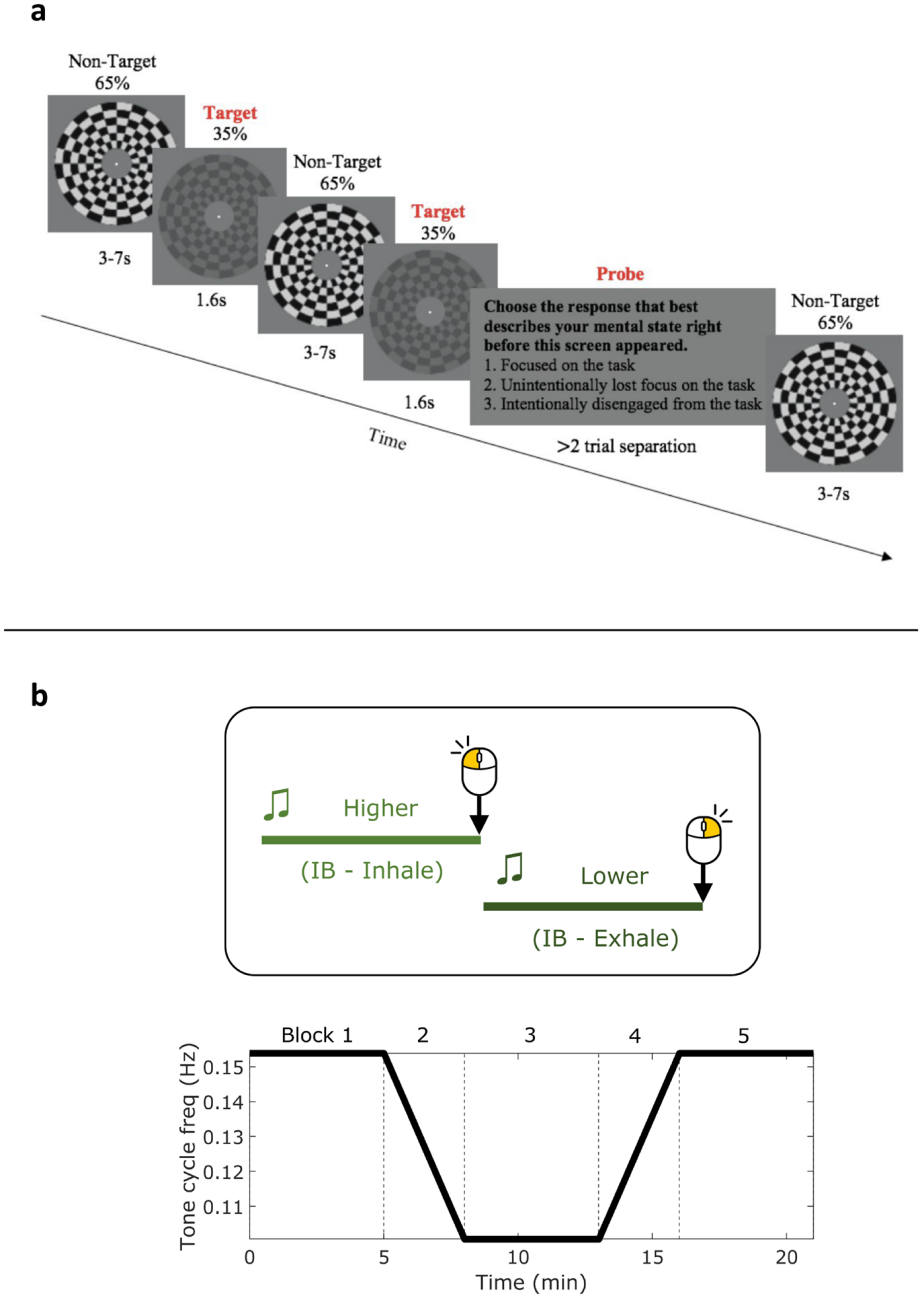
(a) Schematic of the Gradual Contrast Change Detection with Experience Sampling (GradCCD‐ES) Task. A continuously presented, checkerboard, anulus stimulus decreases in contrast after variable intervals which is the target to be identified by participants, who respond with a mouse click upon detection. Additionally, the stimulus is interrupted with experience‐sampling thought probes (TP) which ask the participant to categorise their attentional state immediately prior to the TP presentation. Each block consists of 48 targets, 16 TPs and lasts approximately 8 min each. A testing session consisted of 8 blocks. (b) Schematic for the Paced Auditory Cue Entrainment (PACE) task. Participants heard a higher pitched tone and a lower tone cycling continuously for 21 min, both of which they had to rhythmically respond to with mouse clicks so that left clicks landed on the high → low transition and right on the low → high. An “Instructed Breathing” (IB) group had to additionally inhale during the higher tone and exhale during the lower tone. A “No Instructed Breathing” (NIB) group only responded with mouse clicks. The tone cycle frequency changed over time, starting at 0.15 Hz in block 1, progressively slowing to 0.1 Hz in block 2, remaining at 0.1 Hz in block 3, then speeding up to 0.15 Hz in block 4 and then it remained at 0.15 Hz in block 5. Thus, the rate of mouse responses and rate of respiration for IB only changed accordingly.

In the PACE task participants were required to pace their breathing to an auditory cue which changed in frequency over time. We reported that slow‐paced breathing was related to participants committing less task errors than a control group who were breathing naturally in the PACE task (Andrews, Melnychuk, Moran, McGovern, et al. 2025). Firstly, we are curious to investigate whether the breathing intervention had any influence on sigh frequency. Vlemincx et al. (2012b) asked participants to perform various breathing patterns and noted that sigh frequency increased in the period following the exercises. They did not include a slow‐paced breathing pattern and instead included a “rigid” pattern that controlled respiratory volume (low variability) and a “correlated” pattern where respiratory volume was systematically increased and decreased over time (high structured variability), both guided via biofeedback. Our PACE task contains both elements but for respiratory rate: blocks where rate was constant and blocks where rate was increased and decreased systematically (Figure 1b). For Vlemincx et al. (2012b), sigh frequency was primarily predicted by the increased effort and tension that these imposed breathing patterns induced compared to spontaneous breathing. Qualitative assessments of our participants did not indicate discomfort or difficulty following the breath guide and objective measures indicated high compliance. Due to this, and our lack of biofeedback, our data may not be directly comparable. However, as the only study thus far to investigate imposed breathing patterns and sigh behavior, we hypothesize that our guided breathing group will show a higher number of sighs than the spontaneous breath group due to the imposed “unnatural” breath pattern. We also have behavioral responses within this dataset which can be tested pre‐ to post‐sigh as discussed above.

A foundation to the present research is the possibility that respiration and attention are coupled via the LC‐NA system (Melnychuk et al. 2018, 2021). It has been seen in mice that activation of beta‐noradrenergic receptors in the preBötzinger complex specifically induces sigh behavior, leaving spontaneous respiration uninfluenced (Viemari et al. 2013). It has not yet been tested whether changes in noradrenergic activity relate to sigh behavior in humans. In humans, LC‐NA activity can be accessed using pupil diameter (PD) as a proxy measurement (Bang et al. 2023; DiNuzzo et al. 2019; Elman et al. 2017; Meissner et al. 2023; Murphy et al. 2014), and we have continuous PD tracking in our datasets. We predict that our monotonous, low‐demand tasks will have induced low arousal states in participants and thus sighs may act to increase arousal, which should be concomitant with an increase in PD. This would also be consistent with the finding that PD closely follows respiration depth (Kluger et al. 2023; Ohtsuka et al. 1988), and a sigh has a larger than normal volume.

## 2 Methods

Analysis for the present study on sigh behavior was performed on data acquired for two separate larger analyses (Andrews, Melnychuk, Moran, McGovern, et al. 2025; Andrews, Melnychuk, Moran, Walsh, et al. 2025). In dataset Grad (Andrews, Melnychuk, Moran, Walsh, et al. 2025), participants comprised younger adult (18–35 y/o; *n* = 38) and older adult (65–80 y/o; *n* = 34) groups, who performed 8 blocks of a sustained attention task—the Gradual Contrast Change Detection—Experience Sampling Task (Figure 1a). Participants had to respond with a mouse click when the onscreen stimulus diminished in contrast, as well as responding to thought probes (TP) which assessed their subjective task focus versus mind wandering. Contrast changes (targets) occurred after pseudo randomly variable inter‐target intervals of 3, 5, or 7 s, occurring 48 times per 8 min block. TPs occurred with the same variable pseudorandom intervals (3, 5, or 7 s); however, they only occurred 16 times per block (> 2 target trial separation). For the purposes of the present analysis, age groups were collapsed, and analyses were performed on the whole sample. This was justified since when performing each analysis separately on each group, the statistical conclusions were the same, and thus we took advantage of the increased power from a greater sample size.

In dataset PACE, participants performed the Paced Auditory Cue Entrainment (PACE) task (Figure 1b). Note, the present dataset includes participants enrolled in “Experiment 1” only (and not “Experiment 2”) from Andrews, Melnychuk, Moran, McGovern, et al. (2025). The “Instructed Breathing” (IB) group (*n* = 25) for this study were instructed to respond to auditory tone stimuli with rhythmic mouse clicks, and additionally, use the tone stimuli as a slow‐paced breathing (0.1–0.15 Hz) guide. The “No Instructed Breathing” (NIB) group (*n* = 32) was instructed only to respond to the tones with mouse clicks. Analysis for the present study was performed on these groups separately due to the breath manipulation, and it is clearly stated whether the analysis pertains to the IB or NIB group.

All participants were included in analyses examining between‐subject relationships, including participants with zero detected sighs, as sigh absence constitutes a meaningful outcome of the breathing manipulation.

For pre‐ and post‐sigh analyses, only participants exhibiting at least one detected sigh were included, as within‐sigh comparisons are undefined in the absence of sigh events. Participants without detected sighs were therefore excluded from these analyses only.

## 3 Data Analysis

### 3.1 Respiration Acquisition and Preprocessing

Recording and preprocessing: Respiratory signal was collected using a SleepSense respiratory effort sensor belt placed approximately at the lower portion of the sternum, recorded at 256 Hz via a Biosemi Amplifier and Biosemi Actiview software. Data was preprocessed using custom‐made MATLAB scripts. Signals were first inspected by eye for poor recording sessions and then were low‐pass filtered using a zero‐phase digital Butterworth filter with cutoff frequency 0.6 Hz and filter order 4. Finally, filtered signals were then linearly detrended and *z*‐score normalized within each participant.

### 3.2 Respiratory Variability and Sigh Detection

Respiratory rate (RR) was calculated as 60 divided by the time duration between each breath to obtain a value for each breath. Inspiratory volume (Vi) was the positive peak value minus the preceding trough for each breath. Peaks and troughs were determined on participant‐normalized respiratory data using MATLAB function findpeaks() with a minimum peak width of 0.5 s. The total variability of RR (RR‐CV) and Vi (Vi‐CV) was calculated as the coefficient of variation of the array of values for each. The structured variability was calculated as the coefficient of the lag‐1 autocorrelation for RR (RR‐AR) and Vi (Vi‐AR) by first removing the mean from each series and then calculating the normalized product of adjacent values. The calculation is described in the following formula:

$$\mathrm{AC}\left(1\right)=\frac{\sum_{i=1}^{N-1}\left(x_{i}-\bar{x}\right)\left(x_{i+1}-\bar{x}\right)}{\mathrm{var}\left(x\right)\cdot \left(N-1\right)}$$

Here, 𝑥 = RR or Vi series, 𝑥̄ = series mean, 𝑁 = number of elements in series. Positive values indicate that consecutive breaths tend to be similar (i.e., structured or persistent variability), while negative values reflect alternating or compensatory patterns. An AR value near zero suggests unstructured or random variability.

Random variability in these measures was inferred as the portion of total variability (CV) not accounted for by structured, sequential dependencies (AR). For example, an increase in CV accompanied by a decrease in AR suggests greater unstructured, or “random”, variability in the respiratory signal. Sighs were detected as breaths that had a Vi of at least twice the mean Vi for the block (dataset Grad) or task (dataset PACE) (e.g., Vlemincx et al. 2015).

For the single report of mean respiratory frequency in the first Results section, mean respiratory frequency was calculated using a phase‐based instantaneous frequency measure derived from the Hilbert transform of the filtered respiration signal. The instantaneous phase was unwrapped and its rate of change over time was computed and averaged within each analysis window, before conversion to breaths per minute. This approach provides a reliable estimate under non‐stationary breathing conditions. All other analyses used the methods described above.

### 3.3 Pupillometry Acquisition and Preprocessing

Pupil diameter (PD) was collected using the Eyelink 1000 SR Research camera and associated software from the left eye at 1000 Hz. Participants were instructed to maintain their heads on a chinrest to minimize pupil detection loss. A calibration and validation check was performed before each block for the GradCCD‐ES task and only before the task for the PACE task.

Data were preprocessed using custom made MATLAB scripts. For blink detection, the blink indices provided by the Eyelink automatic detection were extended 50 ms either side. These periods were removed and interpolated linearly. Blink/interpolated sample points within a recording session were saved and used for trial rejection in subsequent analyses, excluding trial windows that contained > 30% blink/interpolated samples. PD was then low pass filtered using a zero‐phase digital Butterworth filter with cut‐off frequency 4 Hz and filter order 4, linearly detrended, and z‐score normalized within each participant.

### 3.4 Pupil Diameter Over Sighs

For both datasets, PD was extracted 3000 ms either side of each sigh peak and also for an equal number of non‐sigh breaths within the same block (Grad) or task (PACE). For selection of non‐sigh breaths, the sigh breaths were removed from the series of peak detections and then a number of breaths were randomly chosen using the MATLAB function randperm() to match the number of detected sighs. PD was then baseline corrected by subtracting the average of the period 500 to 100 ms prior to the window beginning and grand‐averaged across sighs and participants.

### 3.5 Pre‐ and Post‐Sigh Differences

In both datasets we tested for differences in the respiratory variability measures pre‐ and post‐sigh. RR and Vi series were subject to a moving window analysis, calculating CV and AR over a window size of 3 breaths and a moving step size of 1 breath. Means were then taken for the 3 resultant values preceding and proceeding each detected sigh. Reaction time (RT) differences were assessed from the 3 targets prior and post each sigh. In dataset Grad, we additionally assessed changes in the thought probe (TP) responses for a single thought probe each side of each sigh. TPs were categorized into “Focus” (having task‐related thoughts) and “Mind Wandering” (task‐unrelated thoughts), and for each participant we obtained an overall Focus:Mind Wandering ratio pre‐ and post‐ sighs based on these responses. For this analysis participants who provided less than 10 mind wandering responses were excluded.

Therefore, respiratory variability was captured for 7 “unique” breaths (9 minus 2 overlap) before and after each sigh, RT was captured for 3 trials (average 5 s each trial) before and after each sigh, and attentional engagement was assessed for 1 TP before and after each sigh, capturing approximately the 30 s periods either side of them.

### 3.6 Respiratory‐Task Phase‐Locking

In our datasets we used a measure which represented the degree of respiratory phase‐locking between the participants' respiratory cycle and the presentation of task events. Preprocessed respiratory signal for the block was Hilbert transformed to give instantaneous phase information. The phase angles in radians were extracted at the times of task events of interest. These event‐locked phase angles were collapsed across all blocks and the resultant vector length of respiratory phase angles was the primary measure of the strength of respiratory phase‐locking. The vector length is a measure of variability in the respiratory phases occurring at the time of targets, where a higher vector length implies less variability. In dataset Grad, we considered the respiratory phase consistency at the time of contrast changes, the target for response. In dataset PACE we considered the vector length at the beginning times of auditory tone presentation, the targets for response. Both are referred to as “target vector length”. For clarity, if targets occurred at the exact same respiratory phase every single time, the coalesced phases would result in a target vector length of 1, no variability. If targets occurred at random phases, distributed perfectly around the respiratory cycle, this would result in a target vector length of 0, absolutely variable. See Andrews, Melnychuk, Moran, McGovern, et al. (2025), Andrews, Melnychuk, Moran, Walsh, et al. (2025) for the full respiratory phase‐locking analyses. Circular statistics were performed using CircStat toolbox (Berens 2009). See Cremers and Klugkist (2018) for more information on calculating circular statistics.

### 3.7 Statistics

Correlations between sigh frequency and respiratory variability measures were calculated using Pearson's correlation tests. Testing for differences in sigh frequency and respiratory variability measures across task blocks was performed using repeated measures analysis of variance (RM‐ANOVA). Some RM‐ANOVA tests benefitted from Greenhouse–Geisser corrections where an assumption of sphericity was violated (Mauchly's test *p* < 0.05). Post hoc comparisons between blocks were performed where appropriate using the Holm correction method. Pre‐ to post‐ sigh tests were performed using paired Student's *t*‐tests. Circular statistics for respiratory phase‐locking calculations are described in the previous section “Respiratory‐Task Phase‐Locking in dataset Grad”. PD amplitudes for sigh versus non‐sigh breaths were compared for significant differences at time points across grand‐averaged sigh versus non‐sigh breaths by running *t*‐tests at *α* = 0.05.

## 4 Results

It shall be clearly stated whether each analysis pertains to dataset Grad or dataset PACE. Within dataset PACE, there is also the distinction of the “Instructed Breathing” (IB) and “No Instructed Breathing” (NIB) groups, the former of which underwent a slow‐paced breathing intervention during the task.

### 4.1 Sigh and Mean Respiratory Frequency

In dataset Grad (8 × 8 min blocks), there was a mean sigh frequency of 0.63 ± 0.06 sighs per minute. In dataset PACE (21 min task), there was a mean of 0.45 ± 0.07 for the NIB group and 0.17 ± 0.05 for the IB group. The NIB group exhibited significantly more sighs than the IB group, *t*(55) = 3.26, *p* = 0.002. Mean respiratory frequency for dataset Grad was 14.83 ± 0.42 breaths per minute. For dataset PACE NIB group it was 16.28 ± 0.55 and for IB group it was 8.29 ± 0.25.

### 4.2 Sigh Frequency Correlations With Respiratory Variability

**FIGURE 2:**
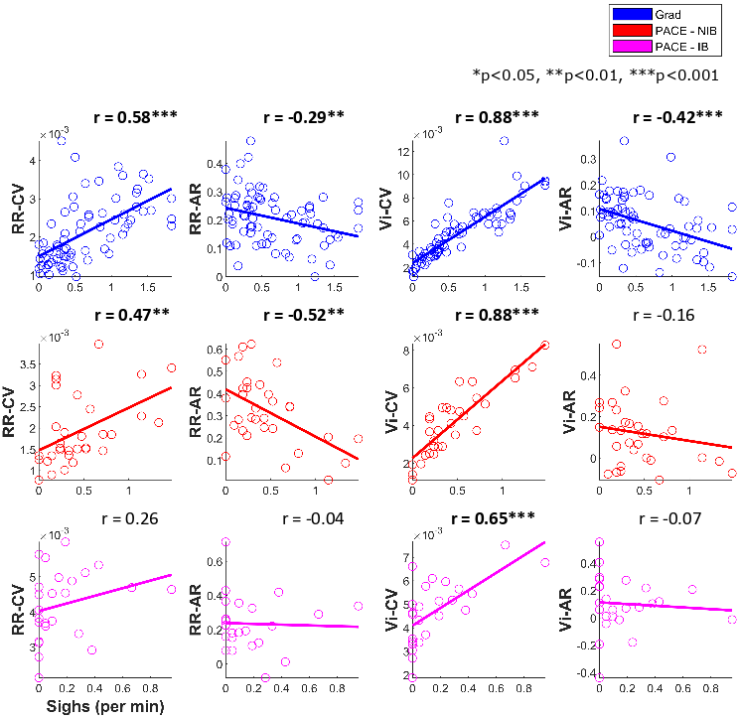
Scatter plots between respiratory variability dynamics and mean sigh counts for each block for dataset Grad (blue, top), and total count for the task for dataset PACE—NIB group (red, middle), IB group (magenta, bottom). Scatter plots between respiratory variability dynamics and mean sigh counts for each block for dataset Grad (blue, top), and total count for the task for dataset PACE—NIB group (red, bottom). Dots represent participant means, and the line represents the line of best linear fit. Overlaid on each plot is the correlation coefficient from Pearson's correlation test between the variables. *p < 0.05, **p < 0.01, ***p < 0.001. AR, autocorrelation; CV, coefficient of variation; RR, respiratory rate; Vi, inspiratory volume.

See Figure 2 for plots showing linear Pearson's correlations between respiratory dynamic variables respiratory rate (RR) and inspiratory volume (Vi) and their total variability (CV) and structured variability (AR) and sigh frequency. In dataset Grad, sigh frequency was significantly positively correlated with RR‐CV *r*(70) = 0.58, *p* < 0.001 and Vi‐CV *r*(70) = 0.88, *p* < and, significantly negatively correlated with RR‐AR *r*(70) = −0.29, *p* = 0.01 and Vi‐AR *r*(70) = −0.42, *p* < 0.001.

In dataset PACE, for group NIB, sigh frequency was significantly positively correlated with RR‐CV *r*(30) = 0.47, *p* = 0.007, and Vi‐CV *r*(30) = 0.88, *p* < 0.001, and significantly negatively correlated with RR‐AR *r*(30) = −0.52, *p* = 0.002 but not with Vi‐AR *r*(30) = −0.16, *p* = 0.37.

For group IB, sigh frequency was not significantly correlated with RR‐CV *r*(23) = 0.26, *p* = 0.21 but was significantly positively correlated with Vi‐CV *r*(23) = 0.65, *p* < 0.001. Sighs were not significantly correlated with RR‐AR *r*(23) = −0.04, *p* = 0.87 nor with Vi‐AR *r*(23) = −0.07, *p* = 0.73.

### 4.3 Sigh Frequency and Respiratory Variability Over Blocks

**FIGURE 3:**
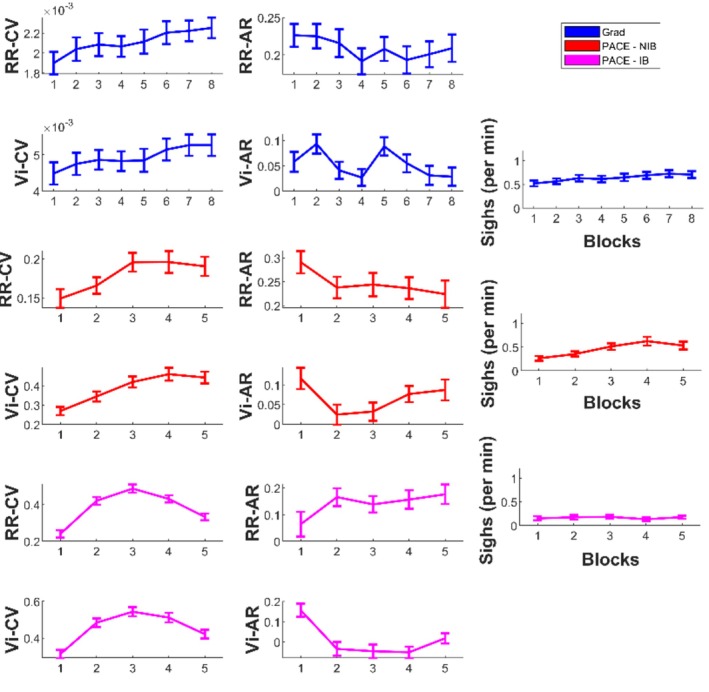
Respiratory variability dynamics and sigh counts over task blocks. Grad in blue, PACE—NIB in red, PACE—IB in magenta. Error bars indicate standard error of the mean. AR, autocorrelation; CV, coefficient of variation; RR, respiratory rate; Vi, inspiratory volume.

See Figure 3 for plots of sigh frequency and respiratory dynamics across the tasks' duration. As per repeated measures analysis of variance (RM‐ANOVA), in dataset Grad, sigh frequency showed a fairly linear trend of increasing across the task blocks, *F*(5.11) = 4.88, *p* < 0.001. Post hoc comparisons showed significant differences between block 1 and blocks 5, 6, 7, 8, all *p* ≤ 0.03. Total variability measures showed significant changes over the blocks, RR‐CV *F*(5.47) = 5.07, *p* < 0.001, Vi‐CV *F*(5.29) = 7.16, *p* < 0.001, showing an increase across blocks. Post hoc comparisons for RR‐CV showed significant differences between block 1 and blocks 6, 7, 8 (*p* ≤ 0.009). Vi‐CV showed significant differences between block 1 and blocks 6, 7, 8 (*p* < 0.001); block 2 and blocks 7, 8 (*p* ≤ 0.015). Structured variability measure of rate did not show significant block‐wise changes, RR‐AR *F*(7) = 0.96, *p* = 0.46, but Vi‐AR did, *F*(5.95) = 0.84, *p* = 0.006, with no significant differences between post hoc comparisons.

In dataset PACE, group NIB, a RM‐ANOVA for sigh frequency across task blocks showed a significant difference across blocks, *F*(3.09) = 4.06, *p* = 0.009, showing a general linear increase over time. Post hoc comparisons showed a significant difference between blocks 1 and 4 (*p* = 0.006). Total variability measures showed significant changes over the blocks with an increase over time: RR‐CV *F*(2.88) = 4.25, *p* = 0.008, post hoc comparisons significant between block 1 and blocks 3, 4, 5 (*p* ≤ 0.04); Vi‐CV *F*(3.17) = 11.82, *p* < 0.001, differences between block 1 and 2, 3, 4, 5 (*p* ≤ 0.001); block 2 and block 4, 5 (*p* < 0.02). RR‐AR did not show significant differences, RR‐AR *F*(3.04) = 0.80, *p* = 0.50, nor did Vi‐AR, *F*(3.08) = 1.74, *p* = 0.16.

For group IB, sigh frequency did not show a significant difference across blocks, *F*(3.21) = 0.24, *p* = 0.88. RR‐CV showed an “inverted *U*‐shaped” pattern across blocks, peaking at block 3, RR‐CV *F*(3.53) = 13.74, *p* < 0.001. Post hoc tests showed significant differences for block 1 versus blocks 2, 3, 4 (*p* < 0.001); block 3 and 5 (*p* < 0.001). Vi‐CV also showed an inverted‐U, Vi‐CV *F*(4) = 14.15, *p* < 0.001, driven by significant differences between block 1 and the higher values in proceeding blocks (post hoc block 1 vs. 2, 3, 4, 5 all *p* < 0.01). There was no significant difference for RR‐AR *F*(4) = 0.68, *p* = 0.60, but there was for Vi‐AR, *F*(4) = 4.85, *p* = 0.001, post hoc differences for block 1 and blocks 2, 3, 4 (*p* < 0.01).

### 4.4 Respiratory Dynamics Pre‐ and Post‐Sigh

**FIGURE 4:**
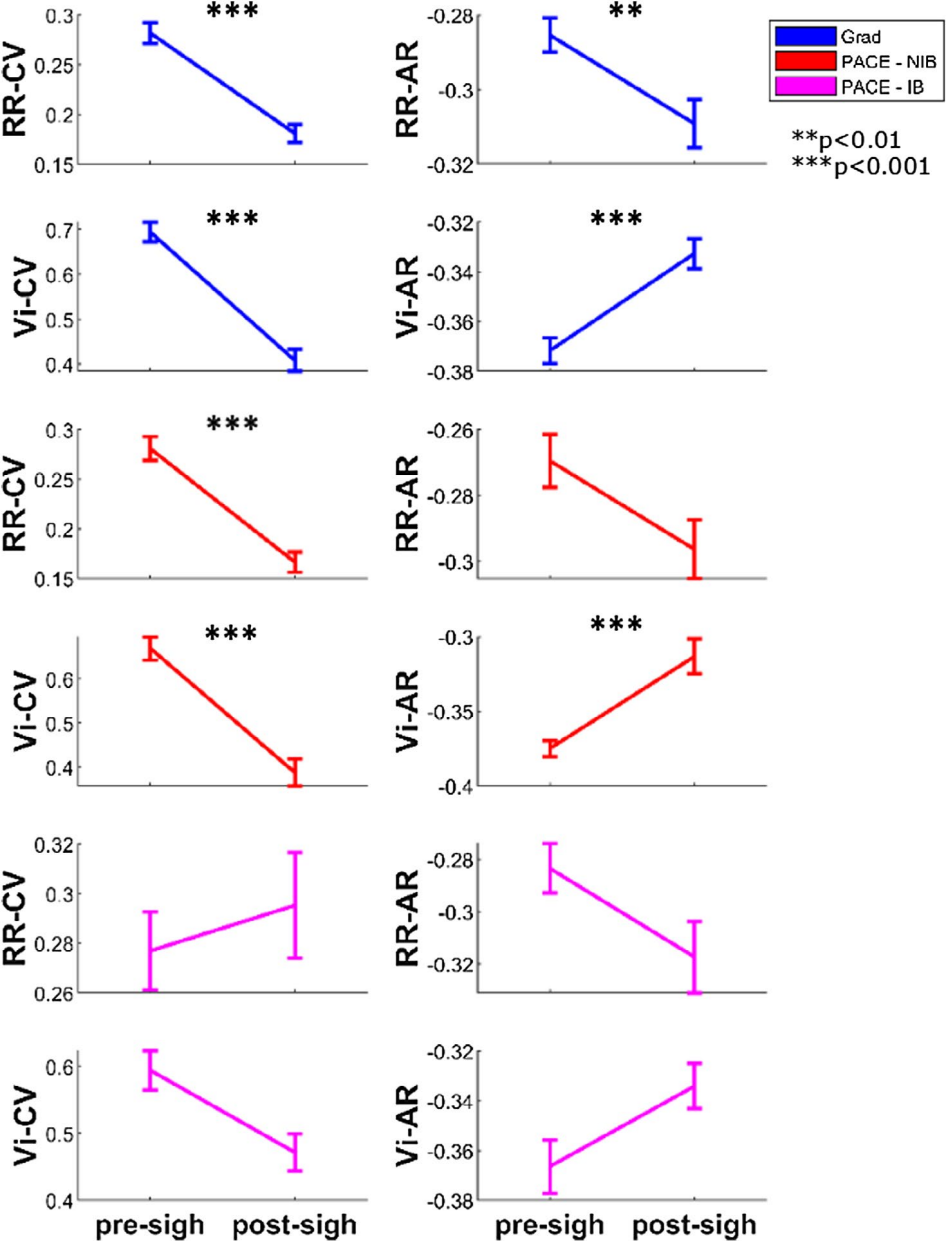
Respiratory dynamics pre‐ and post‐sigh. Error bars indicate standard error of the mean. Top shows dataset Grad (blue), middle is dataset PACE group NIB (red), and bottom shows group IB (magenta).   **p < 0.01, ***p < 0.001. AR, autocorrelation; CV, coefficient of variation; RR, respiratory rate; Vi, inspiratory volume.

See Figure 4. In dataset Grad, total variability measures significantly decreased pre‐ to post‐sigh, RR‐CV *t*(69) = 10.53, *p* < 0.001 and Vi‐CV *t*(69) = 12.02, *p* < 0.001. RR‐AR significantly decreased, RR‐AR *t*(69) = 3.30, *p* = 0.002, and Vi‐AR significantly increased, Vi‐AR *t*(69) = −5.93, *p* < 0.001.

In dataset PACE, for the NIB group, RR‐CV significantly decreased, RR‐CV *t*(27) = 6.28, *p* < 0.001, as did Vi‐CV, *t*(27) = 7.27, *p* < 0.001. No significant difference for RR‐AR *t*(27) = 1.63, *p* = 0.12, but significant increase for Vi‐AR, *t*(27) = −4.12, *p* < 0.001.

The IB group did not show significant differences for any measure, RR‐CV *t*(14) = −0.37, *p* = 0.72, Vi‐CV *t*(14) = 2.02, *p* = 0.06, RR‐AR *t*(14) = 1.11, *p* = 0.28, Vi‐AR *t*(14) = −1.34, *p* = 0.20.

### 4.5 Behavior Pre‐ and Post‐Sigh

In dataset Grad, the reaction time mean, RTm *t*(69) = −0.65, *p* = 0.52, and coefficient of variation, RTCoV *t*(69) = 1.32, *p* = 0.20, from the 3 target responses either side of sighs did not significantly differ. Nor did the Focus:Mind wandering ratio for the thought probe response either side of sighs, *t*(53) = −0.97, *p* = 0.34.

In dataset PACE, for the NIB group, there was no significant difference pre‐ to post‐sigh for RTm *F*(27) = 0.62, *p* = 0.54, or RTCoV *t*(27) = −1.70, *p* = 0.10. Nor for the IB group, RTm *F*(14) = −0.47, *p* = 0.64, RTCoV *t*(14) = −0.98, *p* = 0.34.

### 4.6 Sigh Frequency, Respiratory Variability, and Respiratory‐Task Phase‐Locking

**FIGURE 5:**
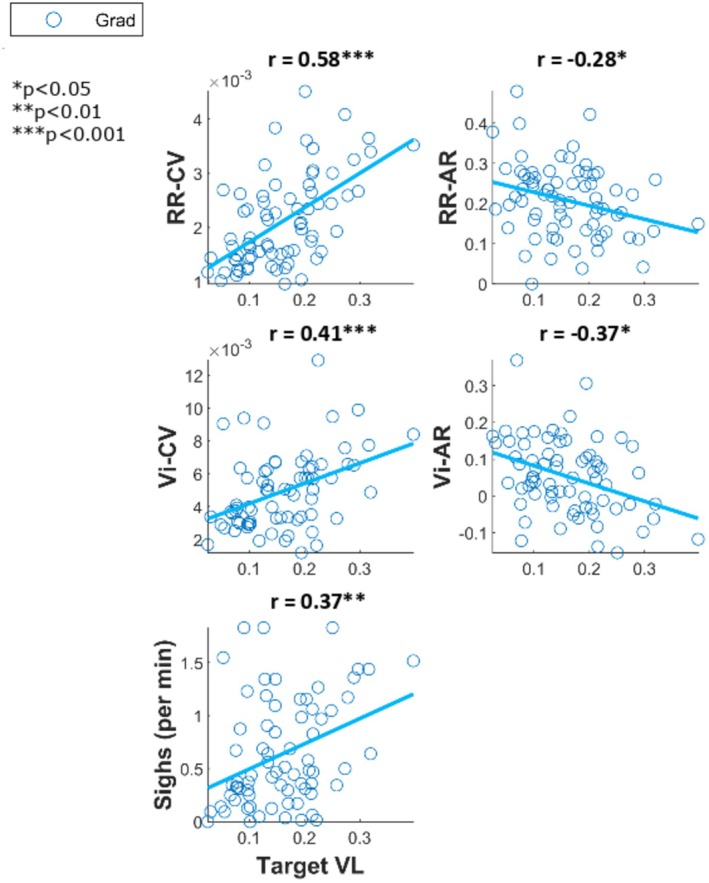
Scatter plots between respiratory dynamics and sigh counts against target vector length (VL, respiratory phase‐locking measure) for dataset Grad. Dots represent participant means and the line represents the line of best linear fit. Overlaid on each plot is the correlation coefficient from Pearson's correlation test between the variables. *p < 0.05, **p < 0.01, ***p < 0.001. AR, autocorrelation; CV, coefficient of variation; RR, respiratory rate; Vi, inspiratory volume.

See Figure 5. In dataset Grad, the degree to which participants phase‐locked their respiratory cycle to the timing of target presentation (target vector length) was significantly positively correlated with sigh frequency *r*(70) = 0.37, *p* = 0.002. Target vector length was positively correlated with RR‐CV *r*(70) = 0.58, *p* < 0.001, and with Vi‐CV *r*(70) = 0.41, *p* < 0.001, significantly negatively correlated with RR‐AR *r*(70) = −0.28, *p* = 0.017 and Vi‐AR *r*(70) = −0.37, *p* = 0.002. In dataset PACE, group NIB, the target vector length was not significantly correlated with any respiratory dynamic measure: sighs *r*(30) = −0.09, *p* = 0.63; RR‐CV *r*(30) = 0.14, *p* = 0.44; Vi‐CV *r*(30) = −0.05, *p* = 0.79; RR‐AR *r*(30) = −0.12, *p* = 0.52; Vi‐AR *r*(30) = 0.09, *p* = 0.62.

### 4.7 Pupil Diameter Over Sighs

**FIGURE 6:**
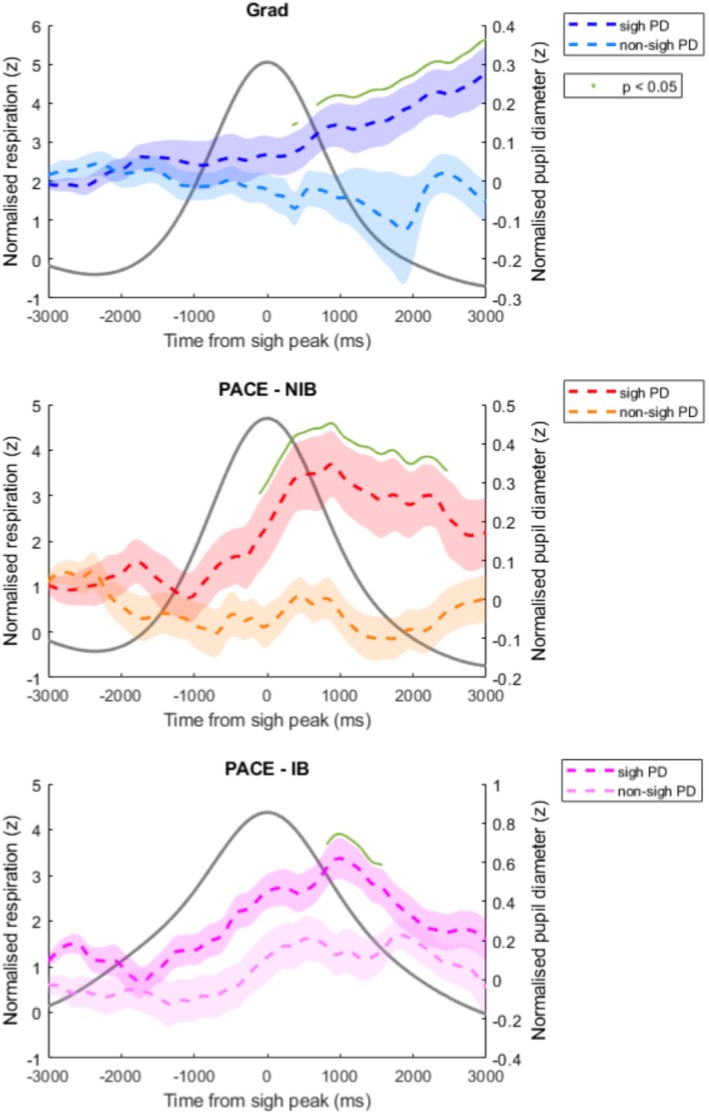
Grand‐averaged, normalized pupil diameter (PD) −3000 ms to +3000 ms with respect to breath peak. PD over sigh shown in darker colours, PD over non‐sigh breaths shown in lighter colours. The grand‐averaged sigh is shown in grey. Shaded areas represent the standard error of the mean (SEM). Green dots above the SEM show significant differences in PD, sigh vs non‐sigh (*p* < 0.05).

Grand averaged pupil diameters (PD) are plotted −3000 to +3000 ms relative to sigh peak and over non‐sigh breaths as a comparison in Figure 6. In dataset Grad, PD over the sigh breaths steadily increased over the entire time course. PD over the non‐sigh breaths stayed fairly consistent. In dataset PACE, for group NIB, PD over sighs showed an initial increase up until the beginning of inhalation, then rising more sharply beyond the respiratory peak, and then steadily decreasing. PD over non‐sigh breaths stayed fairly consistent. For group IB, PD over sighs rose sharply in early‐mid inhalation, peaking just after the respiratory peak and then sharply fell. PD over non‐sighs showed a comparable pattern.

Running *t*‐tests at *α* = 0.05 showed significant sigh versus non‐sigh PD differences over the last third of the sigh duration in dataset Grad, over the latter half of the sigh in NIB, and for a short period around the PD peak approximately 1000 ms post‐sigh peak. It should be noted that these significant time points did not survive Benjamini‐Hochberg FDR correction. However, the high proportion of significant points (Grad: 39.7%, PACE NIB: 42.7%, PACE IB: 12.5%) indicates a temporally extended effect rather than isolated chance fluctuations.

## 5 Discussion

Across two datasets involving distinct sustained attention tasks, we corroborate previous findings demonstrating a robust link between respiratory variability and sigh frequency over time. In groups without explicit breath manipulation, sigh frequency was proportional to the total variability (both random and structured) in respiratory dynamics and tracked these fluctuations across the task duration. Sighs reduced total variability in respiratory rate (RR) and inspiratory volume (Vi), with structured variability showing group‐specific changes, supporting a context‐dependent regulatory role in respiratory dynamics. Applying a slow‐paced breathing manipulation drastically reduced sigh frequency and appeared to mitigate the influence of respiratory variability on sigh occurrence. In the Grad dataset, we previously identified significant phase‐locking between participants' respiration and the timing of pseudo‐randomly presented task targets. Here, we show that the degree of phase‐locking was positively correlated with sigh frequency and with increased random respiratory variability. Finally, we provide the first exploratory evidence in humans linking sighs to concurrent changes in pupil diameter (PD), implicating sigh behavior in the noradrenaline‐mediated arousal system.

Previous work has reported increases in sigh frequency during recovery periods following sustained attention tasks, alongside reductions in respiratory variability (Vlemincx et al. 2011, 2012a). In contrast, during our sustained attention tasks, we observed that sigh frequency increased alongside total respiratory variability. Specifically, sigh frequency rose over time, paralleling a linear increase in CV of both RR and Vi (Figure 3). These increases likely reflect accumulating physiological or cognitive load across the task. Following a sigh, total variability decreased (Figure 4), with RR‐CV and Vi‐CV showing significant reductions, consistent with the hypothesis that sighs reset accumulating variability—potentially mitigating effects of attentional fatigue. In dataset Grad, Vi‐AR increased after a sigh, while RR‐AR decreased, indicating differential effects on volumetric versus rate fluctuations. In dataset PACE, a similar reset in Vi‐AR was observed in the NIB group, but not in the IB group, suggesting that guided breathing attenuated the sigh‐related reset. It should however be noted that our sigh detection protocol would not discriminate between a sigh and a yawn, and increasing yawn frequency expected with increasing fatigue could contribute to the detected “deep breaths.” These observations support the “resetter” perspective on sigh function (Severs et al. 2022; Vlemincx et al. 2022; Vlemincx, Van Diest, et al. 2010).

While structured variability (AR) did not show systematic changes across task blocks (Figure 3), it was nevertheless related to sighing (Figure 2) and showed significant modulation immediately pre‐ and post‐sigh (Figure 4). Specifically, sigh frequency was negatively correlated with AR measures in spontaneous breathing conditions, indicating that sighs tended to occur when respiratory dynamics were less temporally structured. At the same time, AR changed acutely following sighs, with divergent effects for rate and volume. Together, these findings suggest that AR is sensitive to short‐term respiratory dynamics associated with sigh events, rather than reflecting gradual, time‐on‐task changes in variability.

Interestingly, RR‐AR decreased following a sigh in dataset Grad, indicating a transient reduction in the temporal correlation of respiratory rate fluctuations. Rather than contradicting a regulatory role for sighs, this effect can be interpreted in light of the coupling between respiratory dynamics and task timing observed in this dataset. In the Gradual Contrast Change Detection task, targets occurred pseudo‐randomly after 3, 5, or 7 s, yet participants nonetheless synchronized their respiratory cycle to target presentation (Andrews, Melnychuk, Moran, Walsh, et al. 2025). Greater respiratory phase‐locking was associated with higher RR‐CV and lower RR‐AR (Figure 5), indicating that entrainment to irregular external timing increased overall variability while reducing temporal structure. Sigh frequency was also positively related to the degree of phase‐locking. Together, these findings suggest that sighs tend to occur during states of externally driven respiratory entrainment characterized by elevated variability and reduced structure, and that the post‐sigh reduction in RR‐AR may reflect the persistence of this entrained respiratory state rather than a direct reversal of it. Phase‐locking of respiration to task stimuli is increasingly observed (Grund et al. 2022; Huijbers et al. 2014; Johannknecht and Kayser 2022; Melnychuk et al. 2018; Perl et al. 2019). In these studies, respiratory phase‐locking was also concurrent with a respiratory‐phase modulation of cognition or perception, which implicates such phase‐locking in an adaptive function to optimize task performance. It is therefore intriguing to think that whilst this seems to provide a beneficial, adaptive function, the consequence for respiratory variability is a disturbance from the optimal range, which then requires a sigh to reset it. Future research should delve into this relationship further by noting the effect of different inter‐target intervals on respiratory phase‐locking and how this affects sigh frequency. In fact, such a manipulation of task dynamics may serve as a sub‐perceptual manipulation of respiratory dynamic and sigh behavior. No such significant relationships were observed regarding respiratory phase‐locking to the cycling auditory tones in dataset PACE for the “No Instructed Breath” (NIB) group. Phase‐locking in this group was present, though subtle (Andrews, Melnychuk, Moran, McGovern, et al. 2025), but appeared to not have any relation to respiratory variability or sighs. One could have expected that phase‐locking to this consistent temporal rhythm could lower sigh frequency in a reverse of the trend seen in dataset Grad, and echoing the effect of the “Instructed Breathing” group (see proceeding paragraph). However, phase‐locking was likely not a significant factor in the NIB group respiratory dynamics to draw out such an effect.

In the PACE dataset, we analyzed a group following a slow‐paced breathing protocol (IB). Sigh frequency in this group was markedly lower and remained low across the task (Figure 3). Variability in respiratory rate (RR) was strongly constrained by the paced breathing protocol, showing no systematic relationship (Figure 2) with sigh frequency and no significant pre‐ to post‐sigh modulation (Figure 4). Correlations showed sighs in the IB group were positively related to Vi‐CV, implying a link to increased variability in Vi. However, we found no significant pre‐ post‐sigh changes in Vi variability, suggesting that sighs may not have functioned as resets in this context—or that these correlations simply reflect breath amplitude properties of sighs themselves.

A prior study using rigid, biofeedback‐based breathing protocols reported increased sigh frequency during post‐task rest (Vlemincx et al. 2012b) but largely attributed it to elevated tension. The present analyses were restricted to the task period and did not examine post‐task rest, limiting direct comparability with these findings. In contrast, presently, sigh frequency was lower during paced breathing than spontaneous breathing and our participants rated the auditory breath pacing as comfortable. This discrepancy may stem from differences in the pacing method: our tones were designed to mimic soothing breath guides, whereas biofeedback paradigms likely induced more self‐monitoring and stress. Additionally, the PACE task's block structure alternated between fixed (rigid) and gradually changing (correlated) breathing tempos. Sigh frequency remained low throughout, suggesting that this form of control overrode the physiological need for variability resets.

Since sighs are closely linked with stress, it would be curious to investigate their potential role as an indicator of the efficacy of therapeutically oriented breathing exercises and their intended relaxation effects. However, there remains here an ambiguity as to the role of a sigh during this slow‐paced breathing protocol. If they did not appear to track nor reset respiratory dynamics, nor did they appear to indicate a pupil‐linked arousal reset, then what explains their occurrence? Perhaps they did occur presently during infrequent periods where respiratory variability did in fact deviate from homeostatic comfort. Or perhaps there is a “baseline” minimum sigh rhythm generated to ensure, for example, the prevention of alveolar collapse, atelectasis (Severs et al. 2022), or to act as an ultra‐low frequency pacemaker for other currently unknown physiological processes. These speculations should be investigated with future studies applying a variety of guided breath protocols.

We hypothesized that sighs might also reset cognitive states, such as attention or task performance. However, we found no pre‐ to post‐sigh differences in behavioral measures or thought probe responses. Across both datasets, inter‐ and intra‐individual variability in performance was low (Andrews, Melnychuk, Moran, McGovern, et al. 2025; Andrews, Melnychuk, Moran, Walsh, et al. 2025) and participants' self‐reports remained stable. Therefore, any influence of sighing on attentional state may have been too subtle for our task design to detect, or such an effect may only emerge under conditions of greater challenge or arousal. Prior studies reporting subjective relief after sighs typically used stress‐inducing tasks (Vlemincx et al. 2009, 2016; Vlemincx et al. 2010).

Finally, we show for the first time in humans that sighs are concurrent with changes in PD (Figure 6), implicating sighs as part of a noradrenaline‐related arousal mechanism. Sighs have been seen to be noradrenaline‐dependent in mice (Viemari et al. 2013), and the locus coeruleus has been suggested as a key modulator in changing brain states following a sigh (Severs et al. 2022). From a wider perspective, our finding also provides another avenue of evidence for an inherent relationship between respiration and locus coeruleus–noradrenaline (LC‐NA) activity. Preliminary research has demonstrated synchronization between these systems during spontaneous breathing (Andrews, Melnychuk, Moran, Walsh, et al. 2025; Melnychuk et al. 2018, 2021), slow‐paced breathing (Andrews, Melnychuk, Moran, McGovern, et al. 2025), and now during sigh behaviors.

The pattern of PD activity over the sigh was different for each of our groups. In the Grad group, PD steadily rose over the whole period, whereas in the PACE groups, PD rose sharply and then decreased after the sigh peak. Prior analyses on these datasets elucidated that both the stimuli and the breathing intervention highly entrained pupil activity and so this may explain this difference (Andrews, Melnychuk, Moran, McGovern, et al. 2025). The PACE group respiratory‐PD patterns bear strong resemblance to coupling patterns reported recently by Schaefer et al. (2024), replicating this phase‐shift effect. Interestingly, in PACE‐IB PD was modulated similarly whether it was over a sigh or non‐sigh breath. We can speculate therefore that sighing and slow‐paced breathing may recruit similar mechanisms for PD modulation. Given that this respiratory‐pupillary coupling co‐occurred with stable task performance (Andrews, Melnychuk, Moran, McGovern, et al. 2025), it raises the possibility that sighs support dynamic arousal regulation in sustained attention tasks. Further research should examine the causal direction and functional relevance of this coupling.

While our findings provide novel insight into sigh‐related respiratory dynamics, several limitations should be noted. First, sigh detection was based on a trough‐to‐peak amplitude threshold (≥ 2× mean Vi), which may have missed smaller but physiologically relevant sighs or misclassified large breaths, particularly in paced breathing conditions. Second, the cognitive tasks employed (Gradual Contrast Change Detection and PACE) may limit generalizability, as sigh‐respiratory and pupil dynamics could differ under other cognitive or resting‐state conditions. Third, respiration was measured using a single abdominal belt, capturing primarily diaphragmatic volume and potentially underestimating thoracic contributions, especially during task‐related shifts in breathing pattern. Finally, individual differences in baseline physiology, emotional state, and moment‐to‐moment fluctuations may have influenced sigh frequency and respiratory variability and were not fully controlled.

Together, our findings provide compelling evidence that sighs play a dynamic regulatory role in human respiratory variability during sustained attention. Sighs were closely linked to fluctuations in total and random respiratory variability, acted as short‐term resets of respiratory dynamics, and showed associations with arousal‐related physiology. Furthermore, sighs tended to occur during states of externally driven respiratory entrainment characterized by elevated variability and reduced structure. Collectively, this supports the view of sighing as a homeostatic mechanism for maintaining respiratory and cognitive stability during prolonged cognitive engagement. Additionally, our results highlight the modulating effect of slow‐paced breathing on sigh occurrence, offering implications for interventions targeting stress or arousal regulation. Future research should explore how task structure, attentional demands, and paced breathing parameters interact to shape sigh behavior and its functional outcomes.

## Author Contributions

**Ralph W. G. Andrews:** conceptualization, investigation, writing – original draft, methodology, visualization, formal analysis, project administration, writing – review and editing. **Michael C. Melnychuk:** conceptualization, methodology, validation, writing – review and editing, software, resources. **Paul M. Dockree:** conceptualization, funding acquisition, writing – review and editing, methodology, project administration, supervision.

## Funding

This research was funded by the Irish Research Council, grant code: IRCLA/2017/306.

## Ethics Statement

The present study was approved by the Trinity College Dublin, School of Psychology Research Ethics Committee, approval ID: SPREC112020‐16.

## Conflicts of Interest

The authors declare no conflicts of interest.

## Data Availability

The data that support the findings of this study are available from the corresponding author upon reasonable request.
